# Job Demands and Resources Shape the Risk of Burnout in Italian Child Neuropsychiatrists

**DOI:** 10.3390/healthcare13010012

**Published:** 2024-12-24

**Authors:** Alessandra Raspanti, Livio Provenzi, Marta Acampora, Renato Borgatti, Stefania Millepiedi, Isabella L. C. Mariani Wigley, Serena Barello

**Affiliations:** 1 Developmental Psychobiology (DPB) Lab, IRCCS Mondino Foundation, 27100 Pavia, Italy; alessandra.raspanti@mondino.it; 2 Department of Brain and Behavioral Sciences, University of Pavia, 27100 Pavia, Italy; renato.borgatti@unipv.it (R.B.); serena.barello@unipv.it (S.B.); 3 Department of Psychology, Catholic University of Milan, 20123 Milano, Italy; marta.acampora@unicatt.it; 4 Child Neurology and Psychiatry Unit, IRCCS Mondino Foundation, 27100 Pavia, Italy; 5 Department of Child and Adolescent Mental Health, Azienda Usl Toscana Nord-Ovest, 27100 Pisa, Italy; stefania.millepiedi@yahoo.it; 6 FinnBrain Birth Cohort Study, Turku Brain and Mind Center, Department of Clinical Medicine, University of Turku, 20100 Turku, Finland; isabella.marianiwigley@unipd.it; 7 Centre for Population Health Research, Turku University Hospital, University of Turku, 20100 Turku, Finland; 8 Applied Psychology Lab, IRCCS Mondino Foundation, 27100 Pavia, Italy

**Keywords:** burnout, child neuropsychiatrists, demands, mental health, resources, well-being

## Abstract

**Objectives:** To evaluate the influence of job demands and resources on burnout risk among Italian pediatric neuropsychiatrists. **Methods:** This cross-sectional study was conducted between December 2023 and February 2024 and involved Italian pediatric neuropsychiatrists. The study applied the Job Demands-Resources (JD-R) model to assess the impact of job demands (such as work–family conflict, time pressure, and job uncertainty) and job resources (like organizational support and perceived job meaning) on burnout. Burnout was measured through emotional exhaustion, depersonalization, and personal accomplishment subscales. Demographic data, including gender and career stage, were analyzed for their association with burnout. **Results:** High job demands were significantly associated with increased emotional exhaustion and depersonalization, while greater job resources correlated with lower burnout levels and higher personal accomplishment. Gender differences emerged, with female neuropsychiatrists reporting significantly higher emotional exhaustion and lower personal accomplishment than male neuropsychiatrists. Seniority was not a strong predictor, but early-career professionals, particularly residents, exhibited higher susceptibility to emotional exhaustion. Perceived job meaning and organizational support were protective factors across all burnout subscales, buffering the effects of job demands. **Conclusions:** Burnout risk in Italian pediatric neuropsychiatrists is shaped by both job demands and resources. Addressing work–family conflict, job uncertainty, and time pressure alongside enhancing organizational support and fostering job meaning is crucial to mitigate burnout. Special attention should be given to early-career professionals and female neuropsychiatrists to reduce their emotional exhaustion and improve their well-being. These findings provide valuable insights for developing targeted strategies to improve well-being in this field, ultimately enhancing patient care.

## 1. Introduction

The well-being of healthcare professionals is increasingly recognized as a global priority that is crucial to ensuring positive outcomes for patients and their families [1]. Extensive research underscores that healthcare workers in good physical and mental health are more likely to provide high-quality care, which leads to improved patient recovery and satisfaction [2,3]. Moreover, the well-being of healthcare professionals fosters a positive work environment, enhancing teamwork and overall organizational performance [4]. This highlights the interconnectedness between the health of medical staff and the effectiveness of healthcare systems [5].

Despite the recognized importance of healthcare professionals’ well-being, large studies have shown alarming levels of burnout among physicians in recent years. One significant study revealed that in 2014, over 55% of physicians reported at least one symptom of burnout, compared with 46% a few years earlier. Although burnout rates had decreased by 2017, falling back to 2011 levels (44%), depression among physicians increased significantly during the same period, from 38% to 42% [6]. Similar trends have been observed globally, where burnout has been linked to increased absenteeism, higher turnover rates, diminished quality of care, and more frequent medical errors, all of which pose risks to patient safety [7]. The severe impact of burnout on healthcare workers’ professional and personal lives, alongside its economic and public health consequences, has led the World Health Organization (WHO) to include burnout as an occupational phenomenon in the 11th Revision of the International Classification of Diseases (ICD-11) [8]. Addressing burnout is not only crucial for promoting healthcare professionals’ well-being but is also essential for ensuring compliance with legal frameworks such as the European Union’s Framework Directive on Health and Safety (89/391/EEC). This reinforces the need for healthcare systems to evaluate and manage the well-being of their workforce as a priority for both individual and public health [9,10].

The prevalent cultural narrative often portrays healthcare professionals as “invulnerable heroes”, overlooking their psychological vulnerabilities [11]. While this idealized image is intended to emphasize their dedication, it can unintentionally foster a culture of silence, preventing professionals from seeking help for mental health concerns. This, in turn, increases their risk of burnout and other psychological disorders [12]. A more balanced understanding of healthcare professionals, recognizing their human needs and psychological stressors, is therefore essential for developing sustainable and effective interventions to support their well-being.

The COVID-19 pandemic further exacerbated these challenges, with healthcare workers experiencing significant increases in workload, psychological strain, and emotional exhaustion [13]. Numerous studies have shown that the pandemic intensified pre-existing burnout, leading to alarming levels of stress, anxiety, and depression. For instance, research found that 50% of physicians reported burnout during the pandemic, reflecting a 20% increase compared with pre-pandemic levels [14]. In Italy, 80% of healthcare workers reported an increased workload, with 70% experiencing a significant decline in their mental health [15,16]. Resource shortages, emotional strain, and long working hours have compounded the stress on healthcare workers, further emphasizing the urgency of addressing their mental health and well-being [17].

A useful framework for understanding the factors influencing job stress and motivation in healthcare settings is the Job Demands-Resources (JD-R) model developed by Bakker and Demerouti [18]. The model categorizes job stressors into job demands and job resources. Job demands refer to the physical, psychological, social, or organizational aspects of a job that require sustained effort, often resulting in physiological and psychological strain. On the other hand, job resources are the aspects that help employees achieve work goals, reduce job demands, or foster personal development. These resources can serve as intrinsic motivators by promoting employee growth and learning or as extrinsic motivators by helping achieve work objectives. The JD-R model highlights how job stress and motivation function as both outcomes and predictors of job demands and resources, with increased stress and diminished motivation potentially leading to a deterioration in working conditions over time. This model is crucial for developing effective prevention and intervention strategies in the healthcare sector.

Despite the growing recognition of burnout in healthcare, research on the factors contributing to burnout among pediatric neuropsychiatrists remains limited, even though these professionals face particularly high emotional demands in their work [19,20]. Pediatric neuropsychiatrists manage complex cases involving children and adolescents with severe mental health issues such as autism spectrum disorders, ADHD, severe depression, anxiety, and psychosis. These cases often require long-term management and intense interaction with both the young patients and their families, who are often grappling with distress and uncertainty. The emotional burden associated with this level of care is significant, as pediatric neuropsychiatrists must navigate not only the psychiatric conditions of their patients but also the familial and developmental dimensions of each case.

The cumulative stress of managing high caseloads, long working hours, and emotionally intense situations makes pediatric neuropsychiatrists particularly vulnerable to burnout. Their work involves high-stakes decision making, often under conditions of limited resources, which heightens the pressure and potential for emotional exhaustion. Furthermore, the stigma surrounding pediatric mental health can create an isolating environment, where these professionals feel they must meet societal and parental expectations while advocating for proper care for their patients. Additionally, systemic challenges such as insufficient mental health services and underfunding exacerbate the pressures these professionals face, leading to overstretched capacities and an overwhelming workload [21,22,23]. These unique stressors underline the importance of understanding burnout within this specialized field, as addressing these challenges is vital to ensuring the mental health of the workforce and improving patient care.

Given these concerns, the present study seeks to raise awareness about the critical importance of promoting workplace well-being among pediatric neuropsychiatrists in Italy—an under-represented population of pediatric healthcare professionals. Ensuring the health and well-being of these professionals not only is essential for improving their quality of life but is also crucial for enhancing the quality of care provided to children and adolescents. The primary aim of this study is to evaluate how perceived job demands and resources influence the risk of burnout in a large sample of Italian pediatric neuropsychiatrists. Based on previous research, we hypothesize that higher perceived job demands will be associated with an increased risk of burnout, whereas greater perceived job resources will correlate with a reduced risk. The findings from this study are expected to offer practical insights into how workplace conditions can be improved, enhancing both professional well-being and patient care in the field of child and adolescent neuropsychiatry.

## 2. Materials and Methods

### 2.1. Participants

This study was carried out among pediatric neuropsychiatrists between December 2023 and February 2024. An ad hoc questionnaire was developed using the Qualtrics (Provo, UT, USA) platform. The survey link was disseminated through univocal e-mail invitations to members by the Italian Society of Child and Adolescent Neuropsychiatry (SINPIA), and the collected data were stored in a database. To increase the number and diversity of participants, we also employed snowball sampling, a non-probability sampling method, which included individuals who were not members of SINPIA. The study included both resident and specialist pediatric neuropsychiatrists currently employed and working in Italy. Participants who did not provide all the required information in the questionnaire were excluded. Out of 828 potential participants contacted, 676 (82%) agreed to participate. Out of the participants, 144 (17%) did not complete the questionnaires on burnout and psychosomatic symptoms, resulting in a final sample of 532 (64%) responders with complete data. The study was approved by the Ethical Commission of the Department of Psychology—Università Cattolica del Sacro Cuore and adhered to the Declaration of Helsinki. All participants accepted the informed consent prior to starting the online questionnaire.

### 2.2. Procedures

The survey was structured into five main thematic sections: (1) characterization of the socio-demographic and personal profiles of the recruited subjects (including age, gender, nationality, marital status, socio-economic status, profession, clinical area, professional role, work experience, region of work, and number of patients in care); (2) measurement of burnout using the Maslach Burnout Inventory [24]; (3) assessment of psychosomatic symptoms through the administration of the Copenhagen Psychological Questionnaire [25]; (4) measurement of job demands (see Supplementary Materials file), including the following domains based on the model theory: time pressure, work–family conflict, emotional/cognitive overload, uncertainty at work; and (5) measurement of job resources (see Supplementary Materials file), including the following domains: role clarity, meaningful work, organizational support, peer support.

### 2.3. Measures

The survey utilized the Italian adaptation of the Maslach Burnout Inventory [26]. It is a 22-item questionnaire and explores the 3 dimensions of burnout: emotional exhaustion—i.e., feelings of energy depletion (EE; 9 items); depersonalization—i.e., cynicism related to excessive workload (DP; 5 items); and personal accomplishment—i.e., feelings of decreased competence or achievement in one’s work (PA; 8 items). The total score of each dimension was classified as low, moderate, or high according to the cut-offs published by Maslach and Jackson [24]. Participants responded using a 7-point Likert scale ranging from 0 to 6, indicating the frequency with which they had certain types of feelings in relation to their job (levels: 0, never; 1, seldomly in a year; 2, two–three times per month; 3, a few times per month; 4, one time per week; 5, a few times per week; 6, every day).

### 2.4. Plan of Analysis

Descriptive statistics were obtained for job demands and resources as well as for burnout scores and risk bands. Individual differences in job demands and resources linked with gender (i.e., men vs. women) and job seniority (i.e., resident vs. senior) were first explored by means of independent-sample *t*-tests. Differences in the burnout risk classification in the distribution of key output variables were explored by means of chi-squared tests. Multiple hierarchical linear regression models were adopted to separately assess the linear association of job demands (i.e., work–family balance, emotional and cognitive burden, job uncertainty, and time pressure) and resources (i.e., meaning, organizational support, and peer support) with the burnout subscale scores (i.e., emotional exhaustion, personal accomplishment, and depersonalization). In each model, gender and job seniority was included alone in step 1, whereas job demands were added in step 2, and job resources were added in step 3. Outcomes were contrasted with the “high-risk” category as the reference level of the burnout subscale score included in the model as the dependent variable. The results of each hierarchical step are reported extensively in the Supplementary Materials file, whereas the step 3 comprehensive results are described in the full text. The statistical analyses were performed using Jamovi version 2.3.28.0, while plots were obtained from R Studio (version Cranberry Hibiscus, 2024. 9.1.394 for desktop) by means of the ggplot2 package (version 3.5.1; doi: 10.32614/CRAN.package.ggplot2; last access on 15 December 2024).

## 3. Results

### 3.1. Sample Features

**Figure 1:**
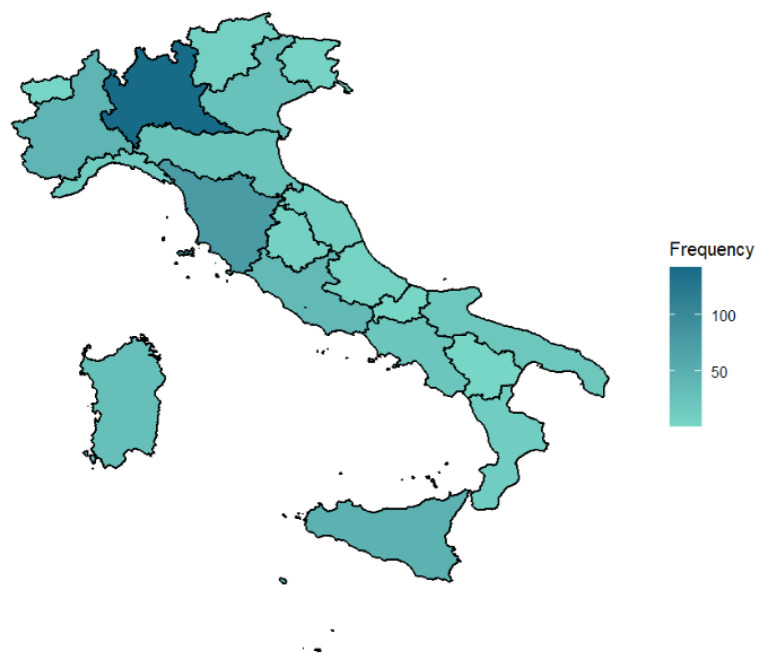
Geographical distribution of study participants.

Among the respondents, 445 participants (84%) were female and 87 (16%) were male. Residents made up 17% of the sample and the rest (83%) were senior. The mean age of responders was 46.8 years (SD = 12.5, range [25.0:74.0]), whereas their seniority ranged from 4 to 50 years (mean = 18.5 years, SD = 11.9). The distribution of the sample by Italian region is reported in Figure 1.

**Figure 2:**
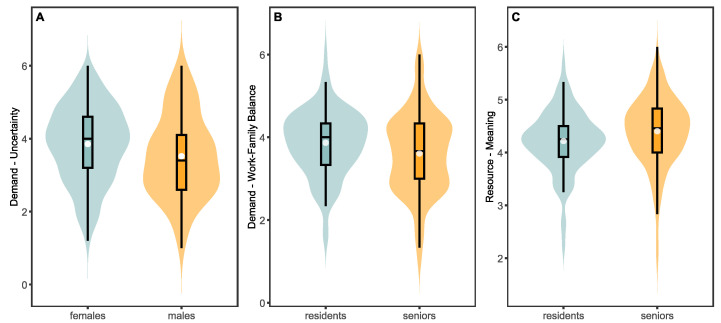
(**A**) Comparison of job uncertainty perception between male and female participants. (**B**) Comparison of work–family conflict perception between resident and senior participants. (**C**) Differences in job meaning perception between resident and senior participants.

Individual differences in job demands and resources between male and female physicians were examined using an independent-sample *t*-test. In terms of job resources, the only significant difference observed was related to job uncertainty, *t*(530) = 2.67, *p* = 0.008 (Figure 2A).

No significant differences were found in work–family conflict (wfami), emotional–cognitive demands (emcog), or time pressure (timep). Moreover, no gender differences were found for the job resources analyzed, although a nearly significant difference was observed in the attribution of meaning to one’s work, *t*(530) = 1.79, *p* = 0.073: specifically, this appeared to be lower in female compared with male physicians. Similarly, we assessed whether there were differences in the same variables with respect to job seniority (i.e., residents vs. senior physicians). Regarding the perception of job demands, the comparison between the two groups revealed a difference only in work–family conflict (wfami), *t*(530) = 2.40, *p* = 0.017: this was found to be higher among residents compared with senior physicians (Figure 2B). The comparison of job resources perception between the two groups revealed statistically significant differences in mean values for job meaning (meani), *t*(530) = 2.52, *p* = 0.012. Specifically, job meaning appeared to be lower among residents compared with senior physicians (Figure 2C).

A similar trend was observed for the perception of personal support (peesu) among residents, although it did not reach statistical significance, *t*(530) = 1.84, *p* = 0.065. No differences were detected for organizational support (orgsu).

### 3.2. Risk Categorization of Burnout Among Italian Pediatric Neuropsychiatrists: Subscales Analysis

**Figure 3:**
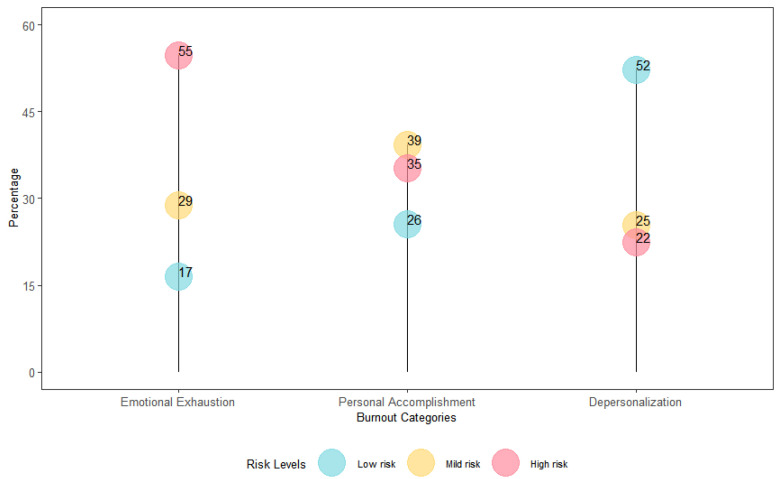
Frequency of different risk categories for the burnout subscales. Data labels represent percentages.

The continuous measurements of the three subscales of the Maslach Burnout Inventory (emotional exhaustion, personal accomplishment, and depersonalization) administered among pediatric neuropsychiatrists have been converted into three categories based on cut-off values: low-, medium-, and high-risk, as previously detailed in the Methods section. Data are presented as the frequency of samples in the three risk categories (low, mild, high), categorized according to the burnout subscales. In Figure 3 are reported frequencies for the emotional exhaustion risk class: 88 (16% of total) show low risk, 153 (29% of total) mild risk, and 291 (55% of total) high risk. In addition, Figure 3 respectively shows the relative frequencies of symptoms associated with the other two subscales, namely, personal accomplishment and depersonalization. For the personal accomplishment subscale, the frequency is the following: 136 (26% of total) low-risk, 209 (39% of total) mild-risk, and 187 (35% of total) high-risk. Finally, we report a low-risk frequency of 278 (52% of total) for depersonalization and frequencies of 209 (39% of total) for mild risk and 187 (35% of total) for high risk.

**Figure 4:**
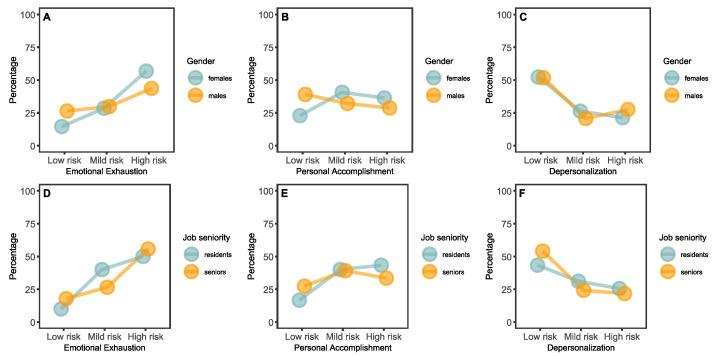
Cumulative frequencies of risk categories for the burnout subscales by gender (**A**–**C**) and job seniority (**D**–**F**).

In Figure 4A, we present the cumulative frequencies of the risk categories related to emotional exhaustion symptoms among female and male respondents. Specifically, 15% (*n* = 65) of female pediatric neuropsychiatrists included in the study reported low risk in this subscale, 29% (*n* = 127) reported a medium risk, and 57% (*n* = 253) reported high risk. Indeed, for male subjects included in the present study, the data revealed that 27% (*n* = 23) showed a low risk of emotional exhaustion, 30% (*n* = 26) a mild risk, while 44% (38) were high-risk. The chi-squared statistics for these data revealed a significant difference between male and female subjects who participated in the study (χ^2^ = 8.51, *p* = 0.014). The analysis of the scores related to symptoms associated with personal accomplishment (Figure 4B) revealed the distribution among female subjects as follows: 22.9% (*n* = 102) were at low risk, 41% (*n* = 181) were at mild risk, and 36% (*n* = 162) were at high risk. For the male responders, we reported: 39% (*n* = 34) low-risk, 32% mild-risk (*n* = 28), and 29% (*n* = 25) high-risk. For emotional exhaustion symptoms, the chi-squared statistics also highlight a significant difference between male and female respondents (χ2 = 9.99, *p* = 0.007). No differences among participants based on job experience was found regarding personal accomplishment and depersonalization (χ^2^ = 2.17, *p* = 0.338) (Figure 4C).

Subsequently, we questioned whether there might be differences in risk categories obtained on the various subscales of the burnout questionnaire based on job seniority. Similarly to gender, we reported a significant difference among residents and seniors for emotional exhaustion symptoms (χ^2^ = 8.00, *p* = 0.018) (Figure 4C). In detail, 10% (*n* = 9) of residents showed a low risk for burnout according to this subscale, 40% (*n* = 36) a mild risk, and 50% (*n* = 45) a high risk. On the contrary, 18% of senior responders (*n* = 79) reported a low risk for emotional exhaustion, 27% (*n* = 117) a mild risk, and 56% (*n* = 246) a high risk. No differences among participants based on job experience was found regarding personal accomplishment and depersonalization (respectively, χ2 = 2.17, *p* = 0.338 and χ^2^ = 3.55, *p* = 0.170) (Figure 4D,E).

### 3.3. Impact of Job Demands and Resources on Burnout: Multiple Hierarchical Linear Regression Model

Subsequently, for each categorical subscale of burnout risk according to the Maslach Burnout Inventory (considered as the dependent variable), we created a three-step multimodal logistic regression model with the aim of (1) verifying whether gender or work experience could serve as significant predictors (step 1), (2) understanding how job demands (i.e., work–family balance, emotional and cognitive burden, job uncertainty, and time pressure) positively correlate with burnout risk (step 2), and (3) investigating the role of job and personal resources (i.e., meaning, organizational support, and peer support) as a protective factor for the effects of elevated demands (step 3). The results obtained from the above-described model will be reported for each of the three subscales of burnout according to the Maslach Burnout Inventory.

**Figure 5:**
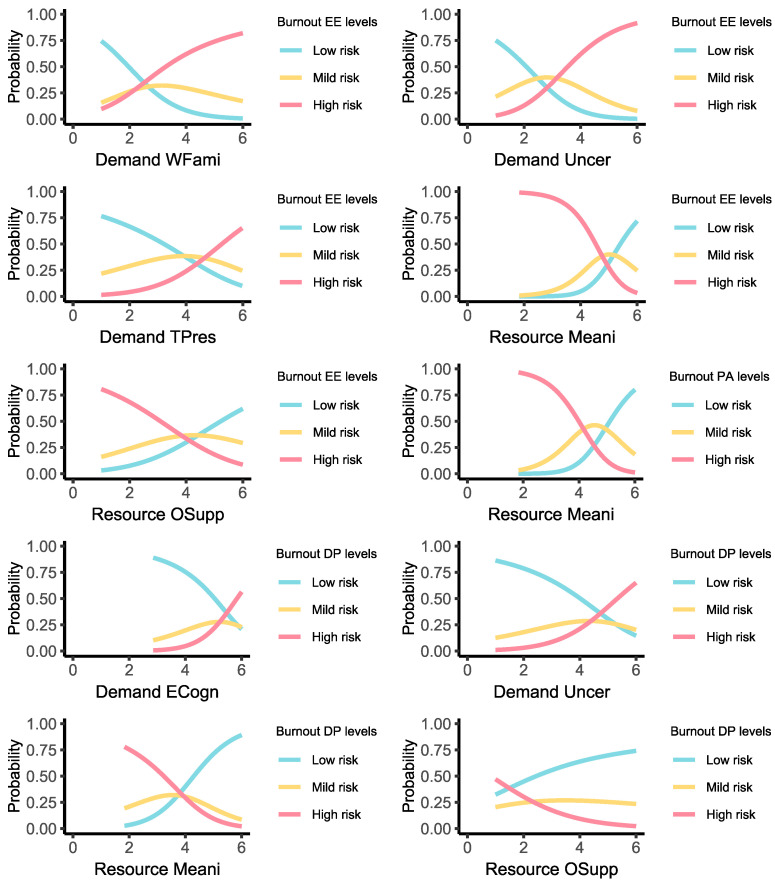
Probability curves illustrating the relationship between job demands and resources and the risk categories of burnout subscales.

For the emotional exhaustion subscale (Figure 5), the final model was statistically significant: R^2^ = 0.28, χ^2^ (18) = 278.70, *p* < 0.001 (Supplementary File). Within this model, the risk of being at high risk for emotional exhaustion vs. being at low risk increased with higher demand_wfami (odds ratio = 0.434, CI 95% [0.29:0.65], *p* < 0.001), higher demand_uncert (odds ratio = 0.357, CI 95% [0.23:0.54], *p* < 0.001), and higher demand_timep (odds ratio = 0.454, CI 95% [0.29:0.70], *p* < 0.001). On the contrary, the following resources seem to have a protective effect, reducing the risk of experiencing high levels of burnout: resource_meani (odds ratio = 3.178, CI 95% [1.45:6.98], *p* = 0.004) and resource_orgsu (odds ratio = 1.663, CI 95% [1.18:2.35], *p* = 0.004). No statistically significant effects emerged for gender and job seniority.

For the personal accomplishment subscale, the final model was statistically significant: R^2^ = 0.16, χ^2^ (18) = 184.00, *p* < 0.001 (Supplementary file). In this model, the likelihood of transitioning from low to high risk of burnout does not appear to be affected by the increase in job demands. On the contrary, an increased perception of job meaningfulness by the responders is associated with an increased probability of low risk of burnout related to the personal accomplishment subscale (odds ratio = 0.103, CI 95% [0.05:0.20], *p* < 0.001) (Figure 5). No statistically significant effects emerged for gender and job seniority.

Finally, we applied the aforementioned three-step model for the last subscale of burnout, the depersonalization category. It was statistically significant: R^2^ = 0.15, χ^2^ (18) = 167.14, *p* < 0.001 (Supplementary File). Unlike the other subscales, we found a significant difference related to gender (i.e., female vs. male) in this case, while no effect was observed for job seniority. As shown in Figure 5, a higher perception of emotional and cognitive demands (demand_emcog) (odds ratio = 2.389, CI 95% [1.32:4.31], *p* =0.004) and job uncertainty (demand_uncert) (odds ratio = 2.115, CI 95% [1.51:2.96], *p* < 0.001) is significantly associated with an increased probability of transitioning from a low to a high risk of depersonalization. Conversely, the presence of internal and external resources such as resource_meani (odds ratio = 0.414, CI 95% [0.24:0.70], *p* < 0.001) and resource_orgsu (odds ratio = 0.628, CI 95% [0.47:0.85], *p* = 0.002), reduces the likelihood of falling into the high-risk category (Figure 5).

## 4. Discussion

The findings from this study provide important insights into the factors contributing to burnout among Italian pediatric neuropsychiatrists, highlighting the nuanced relationships between job demands, job resources, and individual characteristics. These results offer several key implications for understanding burnout in this specialized field and suggest targeted strategies for its mitigation.

One of the most striking findings is the pronounced gender disparity in burnout, particularly in the areas of emotional exhaustion and personal accomplishment. Female pediatric neuropsychiatrists reported significantly higher levels of emotional exhaustion and lower levels of personal accomplishment compared with their male counterparts. These gender differences are consistent with the existing literature, which suggests that female healthcare professionals are more vulnerable to burnout due to a combination of work-related and personal factors [27,28,29]. Women in healthcare often experience higher levels of work–family conflict, which can exacerbate stress and emotional exhaustion. Additionally, gender roles and societal expectations may place additional pressure on women to balance professional responsibilities with caregiving roles at home, further increasing their risk of burnout [9]. This underscores the need for workplace interventions tailored to address the unique challenges faced by female professionals, such as flexible work schedules, family support programs, and mentorship opportunities aimed at fostering work–life balance.

Interestingly, while gender emerged as a significant predictor of burnout, seniority did not play a major role in determining the levels of personal accomplishment and depersonalization. However, early-career professionals, particularly residents, showed higher susceptibility to emotional exhaustion. This aligns with previous research indicating that junior healthcare professionals face heightened burnout risk, often due to the steep learning curve, high pressure, and limited control over their work environment during their training years [30]. Residents may face more intense work–family conflict, time pressure, and uncertainty about their future roles, contributing to emotional strain. This suggests that burnout prevention strategies should particularly focus on early-career professionals, with interventions such as mentoring, peer support, and career guidance to enhance their resilience [31]. These support systems could also address the emotional and psychological burdens residents face as they navigate the demands of clinical practice alongside personal responsibilities.

The application of the Job Demands-Resources (JD-R) model in this study further illuminates the pathways through which burnout develops. High job demands—specifically, work–family conflict, job uncertainty, and time pressure—were significant predictors of emotional exhaustion and depersonalization, reinforcing the model’s assertion that excessive demands are key drivers of burnout [18]. The strong correlation between job uncertainty and burnout is particularly concerning, as it suggests that instability within healthcare systems—whether due to economic pressures, restructuring, or public health crises like the COVID-19 pandemic—can significantly heighten stress levels among pediatric neuropsychiatrists. Uncertainty surrounding job roles, security, and future career progression can create chronic stress, making it difficult for professionals to cope with their day-to-day responsibilities [32]. Therefore, addressing job uncertainty through more transparent communication, secure employment contracts, and clearly defined career paths could be essential to reducing burnout risk in this field.

Conversely, the study also highlights the protective role of job resources, particularly perceived job meaning and organizational support, in reducing burnout risk. The finding that a strong sense of job meaning helps mitigate burnout across all subscales—emotional exhaustion, depersonalization, and personal accomplishment—is a powerful reminder of the importance of fostering a sense of purpose and fulfillment in the workplace. Pediatric neuropsychiatrists, who often deal with emotionally intense cases involving vulnerable young patients, may derive significant job satisfaction from the knowledge that their work has a meaningful and lasting impact on their patients’ lives. This intrinsic motivation can serve as a buffer against the negative effects of high job demands. To promote job meaning, healthcare organizations should focus on creating a supportive and rewarding work environment where professionals feel valued and can see the direct impact of their contributions.

Organizational support also emerges as a critical factor in reducing burnout. This finding aligns with the JD-R model, which posits that job resources such as supervisory support, a positive work culture, and opportunities for professional development can buffer the impact of job demands and promote employee well-being [33]. Pediatric neuropsychiatrists, who often work in high-stress environments, require robust organizational support to prevent burnout and foster long-term resilience. Supportive work environments can include access to psychological services, team-based approaches to managing caseloads, and regular opportunities for professional reflection and feedback. Additionally, fostering a culture of recognition in which the contributions of healthcare professionals are acknowledged and appreciated can play a vital role in enhancing their sense of accomplishment and reducing feelings of depersonalization.

A key limitation of this study relates to issues of generalizability. Although the study aimed to capture a diverse sample of pediatric neuropsychiatrists in Italy, the reliance on a non-probability-based sampling introduces potential biases of self-selection. Similarly, the moderately low participation rate might have further influenced the pool of responses obtained by potential self-selection and requires further replications. At the same time, the theoretical model adopted to guide the hypotheses and analyses provides a robust background to the present findings. Another limitation of this study is its reliance on self-reported measures, which may introduce response biases such as social desirability bias or recall bias. Participants may have underreported or overreported their experiences of burnout, psychosomatic symptoms, or job demands and resources due to personal or professional concerns, particularly given the sensitive nature of these topics. This reliance on subjective reporting limits the objectivity of the data and could affect the accuracy of the associations identified in the analysis.

## 5. Implications

These findings have significant implications for both clinical practice and policy within the field of child and adolescent neuropsychiatry. First, the gender differences in burnout risk point to the necessity of developing gender-sensitive interventions. Healthcare organizations should implement policies aimed at promoting work–life balance, such as flexible working hours and parental leave, while also ensuring that female professionals have equal access to leadership opportunities and career advancement. By addressing these disparities, organizations can help reduce the emotional exhaustion and lower job satisfaction that disproportionately affect female healthcare professionals.

Second, the elevated burnout risk among residents suggests the need for early-career support strategies. Mentorship programs, peer support groups, and structured workload management systems can help alleviate some of the stress experienced by junior professionals. These initiatives can provide early-career healthcare workers with the emotional and professional guidance they need to navigate the challenges of their roles and build resilience over time. Moreover, offering targeted interventions that address the unique stressors faced by residents, such as career uncertainty and high job demands, could help reduce emotional exhaustion and promote a healthier work environment.

Finally, the protective role of job resources, particularly job meaning and organizational support, underscores the importance of cultivating supportive work environments. Healthcare organizations should prioritize creating a culture that values and supports pediatric neuropsychiatrists, offering opportunities for professional growth and development, ensuring manageable workloads, and promoting team-based approaches to patient care. By focusing on these areas, organizations can help reduce the risk of burnout, improve professional satisfaction, and enhance the overall quality of care provided to children and adolescents with mental health needs.

## 6. Conclusions

The study provides valuable insights into the burnout risk factors among pediatric neuropsychiatrists, emphasizing the critical role of job demands, job resources, and individual characteristics. Addressing these factors through targeted interventions, supportive policies, and a culture of recognition and well-being will be essential for reducing burnout and improving both the well-being of healthcare professionals and the care they provide to their patients.

## Data Availability

The data presented in this study are available upon reasonable request from the corresponding author.

## Supplementary Materials

The following supporting information can be downloaded at: https://www.mdpi.com/article/10.3390/healthcare13010012/s1. Table S1. Emotional exhaustion model coefficients—Step 1. Table S2. Emotional exhaustion model coefficients—Step 2. Table S3. Emotional exhaustion model coefficients—Step 3. Table S4. Personal accomplishment model coefficients—Step 1. Table S5. Personal accomplishment model coefficients—Step 2. Table S6. Personal accomplishment model coefficients—Step 3. Table S7. Depersonalization model coefficients—Step 1. Table S8. Depersonalization model coefficients—Step 2. Table S9. Depersonalization model coefficients—Step 3.
