# Supplementary material for: Job Demands and Resources Shape the Risk of Burnout in Italian Child Neuropsychiatrists
Source: Healthcare (Basel). 2024 Dec 24;13(1):12. doi: 10.3390/healthcare13010012 (PMC11720263; doi:10.3390/healthcare13010012)
Supplement: Supplementary file 1 [file healthcare-13-00012-s001.zip › healthcare-3352387-supplementary.pdf]

Supplementary Material for

Job demands and resources shape the risk of burnout in Italian child neuropsychiatrists

Alessandra Raspanti[1], Serena Barello[2,3,*], Marta Acampora[4], Renato Borgatti[2,5], Stefania Millepiedi[6], Isabella L.C. Mariani Wigley[7,8], Livio Provenzi[1,2]

[1] Developmental Psychobiology (dpb) Lab, IRCCS Mondino Foundation, Pavia, ItalyAffiliation 1; e-mail@e-mail.com
[2] Department of Brain and Behavioral Sciences, University of Pavia, Pavia, Italy
[3] Applied Psychology Lab, IRCCS Mondino Foundation, Pavia, Italy
[4] Department of Psychology, Catholic University of Milan, Milano, Italy
[5] Child Neurology and Psychiatry Unit, IRCCS Mondino Foundation, Pavia, Italy
[6] Department of Child and Adolescent Mental Health, ATNO, Pisa, Italy
[7] FinnBrain Birth Cohort Study, Turku Brain and Mind Center, Department of Clinical Medicine, University of Turku, Finland
[8] Centre for Population Health Research, Turku University Hospital and University of Turku
***** Correspondence: serena.barello@unipv.it

1. Hierarchical linear regression models

Multiple hierarchical linear regression models were adopted to separately assess the linear association of job demands (i.e., work-family balance, emotional and cognitive burden, job uncertainty, and time pressure) and resources (i.e., meaning, organizational support, and peer support) with the burnout subscale scores (i.e., emotional exhaustion, personal accomplishment, and depersonalization). In each model, gender and job seniority was included alone in step 1, whereas job demands were added in step 2, and job resources were added in step 3. Outcomes were contrasted with "high-risk" category as the reference level of the burnout subscale score included in the model as dependent variable. The results of each hierarchical step are reported below.

1.1 Emotional exhaustion

							95% Intervallo di Fiducia	
**burnout_emex_category**	**Predittore**	**Stima**	**SE**	**Z**	**p**	**Odds-Ratio**	**Inferiore**	**Superiore**
low risk - high risk	Intercettare	-1.781	0.374	-4.76	< .001	0.168	0.0809	0.351
	gender:							
	males – females	0.861	0.299	2.88	0.004	2.364	1.3156	4.249
	job seniority:							
	Senior – Resident	0.482	0.390	1.24	0.216	1.620	0.7546	3.477
mild risk - high risk	Intercettare	-0.271	0.228	-1.19	0.235	0.763	0.4878	1.193
	gender:							
	males – females	0.304	0.278	1.09	0.274	1.356	0.7862	2.338
	job seniority:							
	Senior – Resident	-0.517	0.251	-2.07	0.039	0.596	0.3648	0.974

Table S1. Emotional exhaustion model coefficients – Step 1.

							95% Intervallo di Fiducia	
**burnout_emex_category**	**Predittore**	**Stima**	**SE**	**Z**	**p**	**Odds-Ratio**	**Inferiore**	**Superiore**
low risk - high risk	Intercettare	12.7307	1.794	7.096	< .001	337949.947	10040.662	1.14e+7
	gender:							
	males – females	0.4340	0.381	1.140	0.254	1.543	0.732	3.256
	job seniority:							
	Senior – Resident	0.4033	0.468	0.862	0.389	1.497	0.598	3.746
	demand_wfami	-0.9454	0.191	-4.951	< .001	0.389	0.267	0.565
	demand_emcog	-0.4632	0.314	-1.475	0.140	0.629	0.340	1.165
	demand_uncert	-1.3893	0.197	-7.044	< .001	0.249	0.169	0.367
	demand_timep	-0.7406	0.214	-3.464	< .001	0.477	0.314	0.725
mild risk - high risk	Intercettare	7.5083	1.416	5.304	< .001	1823.167	113.724	29228.055
	gender:							
	males – females	0.0755	0.302	0.250	0.803	1.078	0.597	1.949
	job seniority:							
	Senior – Resident	-0.4665	0.275	-1.693	0.090	0.627	0.366	1.076
	demand_wfami	-0.3025	0.132	-2.300	0.021	0.739	0.571	0.956
	demand_emcog	-0.1804	0.240	-0.751	0.452	0.835	0.521	1.337
	demand_uncert	-0.7139	0.136	-5.233	< .001	0.490	0.375	0.640
	demand_timep	-0.5388	0.178	-3.032	0.002	0.583	0.412	0.827

Table S2. Emotional exhaustion model coefficients – Step 2. demand_wfami = work-family conflict; demand_emcog = emotional-cognitive demands; demand_uncert = job uncertainty perception; demand_timep = time pressure.

							95% Intervallo di Fiducia	
**burnout_emex_category**	**Predittore**	**Stima**	**SE**	**Z**	**p**	**Odds-Ratio**	**Inferiore**	**Superiore**
low risk - high risk	Intercettare	2.8600	2.653	1.0778	0.281	17.462	0.0963	3167.463
	gender:							
	males – females	0.3829	0.407	0.9408	0.347	1.466	0.6605	3.256
	job seniority:							
	Senior – Resident	0.1000	0.495	0.2021	0.840	1.105	0.4190	2.915
	demand_wfami	-0.8347	0.206	-4.0456	< .001	0.434	0.2896	0.650
	demand_emcog	-0.3405	0.340	-1.0003	0.317	0.711	0.3650	1.386
	demand_uncert	-1.0311	0.213	-4.8324	< .001	0.357	0.2347	0.542
	demand_timep	-0.7897	0.226	-3.4867	< .001	0.454	0.2912	0.708
	resource_meani	1.1562	0.402	2.8786	0.004	3.178	1.4463	6.983
	resource_orgsu	0.5084	0.176	2.8901	0.004	1.663	1.1778	2.347
	resource_peesu	0.3382	0.210	1.6141	0.107	1.402	0.9301	2.114
mild risk - high risk	Intercettare	-0.1111	1.980	-0.0561	0.955	0.895	0.0185	43.392
	gender:							
	males – females	0.0924	0.317	0.2911	0.771	1.097	0.5887	2.043
	job seniority:							
	Senior – Resident	-0.7230	0.295	-2.4524	0.014	0.485	0.2723	0.865
	demand_wfami	-0.2240	0.144	-1.5562	0.120	0.799	0.6028	1.060
	demand_emcog	-0.0314	0.251	-0.1251	0.900	0.969	0.5921	1.586
	demand_uncert	-0.4851	0.148	-3.2799	0.001	0.616	0.4607	0.823
	demand_timep	-0.5636	0.186	-3.0232	0.003	0.569	0.3950	0.820
	resource_meani	1.0304	0.269	3.8310	< .001	2.802	1.6540	4.747
	resource_orgsu	0.2186	0.124	1.7568	0.079	1.244	0.9750	1.588
	resource_peesu	0.2266	0.132	1.7199	0.085	1.254	0.9689	1.624

Table S3. Emotional exhaustion model coefficients – Step 3. demand_wfami = work-family conflict; demand_emcog = emotional-cognitive demands; demand_uncert = job uncertainty perception; demand_timep = time pressure; resource_meani = job meaning; resource_orgsu = organizational support; resource_peesu = perception of personal support.

1.2 Personal accomplishment

							95% Intervallo di Fiducia	
**burnout_persacc_category**	**Predittore**	**Stima**	**SE**	**Z**	**p**	**Odds-Ratio**	**Inferiore**	**Superiore**
mild risk - low risk	Intercettare	1.034	0.316	3.27	0.001	2.811	1.514	5.219
	gender:							
	males – females	-0.776	0.285	-2.73	0.006	0.460	0.264	0.804
	job seniority:							
	Senior – Resident	-0.533	0.332	-1.61	0.108	0.587	0.306	1.125
high risk - low risk	Intercettare	1.115	0.313	3.57	< .001	3.049	1.652	5.627
	gender:							
	males – females	-0.783	0.294	-2.66	0.008	0.457	0.257	0.814
	job seniority:							
	Senior – Resident	-0.769	0.330	-2.33	0.020	0.463	0.243	0.885

Table S4. Personal accomplishment model coefficients – Step 1.

burnout_persacc_category	Predittore	Stima	SE	Z	p	Odds-Ratio	95% Intervallo di Fiducia	
							Inferiore	Superiore
mild risk - low risk	Intercettare	-1.6674	1.170	-1.426	0.154	0.18874	0.0191	1.868
	gender:							
	males – females	-0.6920	0.295	-2.342	0.019	0.50056	0.2805	0.893
	job seniority:							
	Senior – Resident	-0.5667	0.341	-1.663	0.096	0.56741	0.2910	1.106
	demand_wfami	-0.0275	0.131	-0.209	0.834	0.97285	0.7519	1.259
	demand_emcog	0.7218	0.243	2.974	0.003	2.05819	1.2790	3.312
	demand_uncert	0.2675	0.134	1.998	0.046	1.30664	1.0051	1.699
	demand_timep	-0.2950	0.156	-1.890	0.059	0.74455	0.5483	1.011
high risk - low risk	Intercettare	-4.6291	1.368	-3.384	< .001	0.00976	6.69e-4	0.143
	gender:							
	males – females	-0.5428	0.326	-1.664	0.096	0.58109	0.3065	1.102
	job seniority:							
	Senior – Resident	-0.8648	0.354	-2.440	0.015	0.42112	0.2102	0.843
	demand_wfami	0.2607	0.143	1.824	0.068	1.29782	0.9808	1.717
	demand_emcog	0.4472	0.268	1.667	0.096	1.56396	0.9244	2.646
	demand_uncert	0.8568	0.152	5.633	< .001	2.35572	1.7484	3.174
	demand_timep	-0.1119	0.186	-0.602	0.547	0.89416	0.6211	1.287

Table S5. Personal accomplishment model coefficients – Step 2. demand_wfami = work-family conflict; demand_emcog = emotional-cognitive demands; demand_uncert = job uncertainty perception; demand_timep = time pressure.

burnout_persacc_category	Predittore	Stima	SE	Z	p	Odds-Ratio	95% Intervallo di Fiducia	
							Inferiore	Superiore
mild risk - low risk	Intercettare	4.2951	2.028	2.118	0.034	73.336	1.3783	3902.114
	gender:							
	males – females	-0.6697	0.304	-2.202	0.028	0.512	0.2820	0.929
	job seniority:							
	Senior – Resident	-0.3913	0.350	-1.119	0.263	0.676	0.3408	1.342
	demand_wfami	-0.0999	0.141	-0.707	0.480	0.905	0.6860	1.194
	demand_emcog	0.6307	0.254	2.483	0.013	1.879	1.1420	3.091
	demand_uncert	0.0873	0.147	0.594	0.552	1.091	0.8183	1.455
	demand_timep	-0.2844	0.160	-1.775	0.076	0.752	0.5497	1.030
	resource_meani	-1.1541	0.304	-3.799	< .001	0.315	0.1739	0.572
	resource_orgsu	-0.0392	0.124	-0.315	0.752	0.962	0.7539	1.227
	resource_peesu	0.1349	0.141	0.955	0.340	1.144	0.8676	1.510
high risk - low risk	Intercettare	8.5079	2.278	3.734	< .001	4953.800	56.9773	430699.880
	gender:							
	males – females	-0.5796	0.354	-1.636	0.102	0.560	0.2797	1.122
	job seniority:							
	Senior – Resident	-0.5438	0.375	-1.449	0.147	0.581	0.2782	1.212
	demand_wfami	0.0683	0.160	0.427	0.670	1.071	0.7823	1.465
	demand_emcog	0.2501	0.291	0.859	0.390	1.284	0.7258	2.272
	demand_uncert	0.4362	0.170	2.561	0.010	1.547	1.1078	2.160
	demand_timep	-0.1243	0.197	-0.631	0.528	0.883	0.6003	1.299
	resource_meani	-2.2695	0.336	-6.748	< .001	0.103	0.0535	0.200
	resource_orgsu	-0.0693	0.144	-0.480	0.631	0.933	0.7032	1.238
	resource_peesu	0.0335	0.153	0.219	0.827	1.034	0.7666	1.395

Table S6. Personal accomplishment model coefficients – Step 3. demand_wfami = work-family conflict; demand_emcog = emotional-cognitive demands; demand_uncert = job uncertainty perception; demand_timep = time pressure; resource_meani = job meaning; resource_orgsu = organizational support; resource_peesu = perception of personal support.

1.3 Depersonalization

burnout_depers_category	Predittore	Stima	SE	Z	p	Odds-Ratio	95% Intervallo di Fiducia	
							Inferiore	Superiore
mild risk - low risk	Intercettare	-0.297	0.252	-1.179	0.238	0.743	0.454	1.217
	gender:							
	males – females	-0.230	0.302	-0.761	0.447	0.795	0.440	1.436
	job seniority:							
	Senior – Resident	-0.473	0.274	-1.728	0.084	0.623	0.364	1.066
high risk - low risk	Intercettare	-0.577	0.269	-2.151	0.032	0.561	0.332	0.950
	gender:							
	males – females	0.267	0.281	0.950	0.342	1.306	0.753	2.265
	job seniority:							
	Senior – Resident	-0.383	0.290	-1.322	0.186	0.682	0.387	1.203

Table S7. Depersonalization model coefficients – Step 1.

burnout_depers_category	Predittore	Stima	SE	Z	p	Odds-Ratio	95% Intervallo di Fiducia	
							Inferiore	Superiore
mild risk - low risk	Intercettare	-2.8777	1.132	-2.5425	0.011	0.0563	0.00612	0.517
	gender:							
	males – females	-0.0284	0.314	-0.0906	0.928	0.9720	0.52560	1.797
	job seniority:							
	Senior – Resident	-0.5232	0.284	-1.8452	0.065	0.5926	0.33997	1.033
	demand_wfami	0.0586	0.124	0.4728	0.636	1.0603	0.83176	1.352
	demand_emcog	0.4826	0.237	2.0372	0.042	1.6203	1.01847	2.578
	demand_uncert	0.3624	0.129	2.8094	0.005	1.4368	1.11581	1.850
	demand_timep	-0.2401	0.146	-1.6427	0.100	0.7865	0.59059	1.047
high risk - low risk	Intercettare	-10.5025	1.702	-6.1689	< .001	2.75e-5	9.76e-7	7.73e-4
	gender:							
	males – females	0.8063	0.334	2.4126	0.016	2.2396	1.16333	4.312
	job seniority:							
	Senior – Resident	-0.5262	0.329	-1.5982	0.110	0.5908	0.30988	1.126
	demand_wfami	0.2016	0.145	1.3905	0.164	1.2233	0.92076	1.625
	demand_emcog	0.9742	0.296	3.2865	0.001	2.6491	1.48177	4.736
	demand_uncert	0.9241	0.162	5.7210	< .001	2.5195	1.83583	3.458
	demand_timep	0.1084	0.221	0.4895	0.624	1.1145	0.72213	1.720

Table S8. Depersonalization model coefficients – Step 2. demand_wfami = work-family conflict; demand_emcog = emotional-cognitive demands; demand_uncert = job uncertainty perception; demand_timep = time pressure.

burnout_depers_category	Predittore	Stima	SE	Z	p	Odds-Ratio	95% Intervallo di Fiducia	
							Inferiore	Superiore
mild risk - low risk	Intercettare	1.6266	1.714	0.9490	0.343	5.08642	0.177	146.335
	gender:							
	males – females	-0.0138	0.319	-0.0434	0.965	0.98625	0.528	1.844
	job seniority:							
	Senior – Resident	-0.3888	0.290	-1.3430	0.179	0.67785	0.384	1.196
	demand_wfami	-0.0274	0.129	-0.2127	0.832	0.97296	0.756	1.253
	demand_emcog	0.4253	0.242	1.7578	0.079	1.53000	0.952	2.458
	demand_uncert	0.1978	0.139	1.4206	0.155	1.21876	0.928	1.601
	demand_timep	-0.2238	0.149	-1.5011	0.133	0.79949	0.597	1.071
	resource_meani	-0.7633	0.243	-3.1455	0.002	0.46611	0.290	0.750
	resource_orgsu	0.1356	0.121	1.1212	0.262	1.14520	0.904	1.451
	resource_peesu	-0.1262	0.124	-1.0184	0.309	0.88144	0.691	1.124
high risk - low risk	Intercettare	-4.7279	2.244	-2.1068	0.035	0.00885	1.09e-4	0.719
	gender:							
	males – females	0.9226	0.348	2.6515	0.008	2.51577	1.272	4.976
	job seniority:							
	Senior – Resident	-0.4417	0.343	-1.2861	0.198	0.64294	0.328	1.260
	demand_wfami	0.1011	0.153	0.6619	0.508	1.10643	0.820	1.493
	demand_emcog	0.8711	0.301	2.8950	0.004	2.38962	1.325	4.310
	demand_uncert	0.7491	0.172	4.3576	< .001	2.11500	1.510	2.962
	demand_timep	0.0246	0.223	0.1104	0.912	1.02490	0.662	1.586
	resource_meani	-0.8807	0.267	-3.2941	< .001	0.41450	0.245	0.700
	resource_orgsu	-0.4649	0.152	-3.0579	0.002	0.62819	0.466	0.846
	resource_peesu	0.2879	0.146	1.9768	0.048	1.33361	1.002	1.774

Table S9. Depersonalization model coefficients – Step 3. demand_wfami = work-family conflict; demand_emcog = emotional-cognitive demands; demand_uncert = job uncertainty perception; demand_timep = time pressure; resource_meani = job meaning; resource_orgsu = organizational support; resource_peesu = perception of personal support.
